# AMRrounds: susceptibility to the new tetracycline-derivative eravacycline of New Delhi metallo-β-lactamase-producing *Klebsiella pneumoniae* complex

**DOI:** 10.1093/jacamr/dlaf153

**Published:** 2025-08-28

**Authors:** Valentina Galfo, Giusy Tiseo, Cesira Giordano, Alessandro Leonildi, Aurelio Lepore, Manuela Pogliaghi, Niccolò Riccardi, Simona Barnini, Marco Falcone

**Affiliations:** Infectious Diseases Unit, Department of Clinical and Experimental Medicine, Azienda Ospedaliero Universitaria Pisana, University of Pisa, Pisa, Italy; Infectious Diseases Unit, Department of Clinical and Experimental Medicine, Azienda Ospedaliero Universitaria Pisana, University of Pisa, Pisa, Italy; Microbiology Unit, Azienda Ospedaliero Universitaria Pisana, Pisa, Italy; Microbiology Unit, Azienda Ospedaliero Universitaria Pisana, Pisa, Italy; Infectious Diseases Unit, Department of Clinical and Experimental Medicine, Azienda Ospedaliero Universitaria Pisana, University of Pisa, Pisa, Italy; Infectious Diseases Unit, Department of Clinical and Experimental Medicine, Azienda Ospedaliero Universitaria Pisana, University of Pisa, Pisa, Italy; Infectious Diseases Unit, Department of Clinical and Experimental Medicine, Azienda Ospedaliero Universitaria Pisana, University of Pisa, Pisa, Italy; Microbiology Unit, Azienda Ospedaliero Universitaria Pisana, Pisa, Italy; Infectious Diseases Unit, Department of Clinical and Experimental Medicine, Azienda Ospedaliero Universitaria Pisana, University of Pisa, Pisa, Italy

## Case

**Figure 1.**
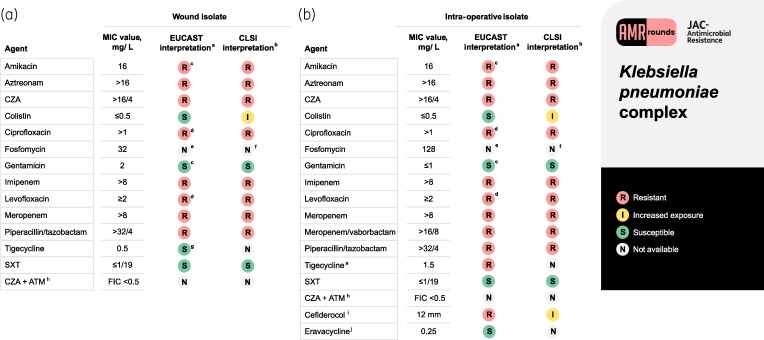
Antibiotic susceptibility profile for wound isolate (a) and intra-operative isolate (b) corresponding to the clinical case. Antimicrobial susceptibility testing was performed with the BD Phoenix (Becton Dickinson) and/or Micronaut (Merlin Diagnostika) systems, according to the manufacturer’s instructions, susceptibility was evaluated as per EUCAST v15.0 breakpoints. MICs for minocycline were not determined. S, susceptible; I, increased exposure; R, resistant; N, not available; ATM, aztreonam; CZA, ceftazidime/avibactam; SXT, trimethoprim/sulfamethoxazole. ^a^According to EUCAST v15.0 clinical breakpoints (2025). ^b^According to CLSI standard M100, 35th Edition (2025). ^c^According to EUCAST v15.0 for systemic infections, aminoglycosides should be combined with other active agents; according to ‘EUCAST breakpoints in brackets’ (December 2021), these breakpoints are provisional and require cautious interpretation. ^d^According to EUCAST v15.0, if resistant to ciprofloxacin, then report as resistant to all fluoroquinolones. ^e^According to EUCAST v15.0, there is currently a lack of clinical evidence to support clinical breakpoints. ^f^According to CLSI 35th Edition, no breakpoints are available except for urinary isolates. ^g^According to EUCAST v15.0, breakpoints are available for *Escherichia coli* and *Citrobacter koseri*. ^h^Checkerboard analysis in Mueller-Hinton broth and synergy defined as a FIC index ≤  0.5. Checkerboards were set up with 2-fold dilutions of aztreonam (1 to 32 mg/L) and ceftazidime/avibactam (0.25/4 to 16/4 mg/L). ^i^Disc diffusion test using 30 mg cefiderocol discs; Liofilchem. ^j^Broth microdilution method, Thermo Scientific^™^ EUMDROXF AST Plate, according to EUCAST v15.0 breakpoint available for *E. coli.*

A 39-year-old male with glucose-6-phosphate dehydrogenase (G6PD) deficiency was admitted to the intensive care unit of the University Hospital of Pisa for critical lower limb ischaemia needing surgical revascularization. On Day 14 from admission, swelling, dehiscence and purulent secretions appeared at the surgical site. A computed tomography scan showed oedema and thickening of subcutaneous soft tissues and muscular structures. Samples from the wound grew a NDM-producing *Klebsiella pneumoniae* complex *(Kp).* The strain carried *bla_NDM_* and *bla_CTX-M_* genes, detected by PCR. Isolate identification and antimicrobial susceptibility tests were performed as previously described.^1^ MIC values were interpreted according to EUCAST breakpoints (Figure 1a). The combination ceftazidime/avibactam *plus* aztreonam showed full synergy with a fractional inhibitory concentration index ≤ 0.5 using checkerboards.^2^ Trimethoprim/sulfamethoxazole was contraindicated because of G6PD deficiency. Colistin was avoided due to elevated creatinine levels (2.72 mg/dL, eGFR 26.2 mL/min/1.73 m²) and concerns about tissue penetration. Thus, tigecycline 50 mg q12h, administered as intermittent infusion, in combination with ceftazidime/avibactam 2.5 g q8h *plus* aztreonam 2 g q8h, given via continuous simultaneous infusion, was started. However, after 10 days of therapy, clinical conditions worsened; the patient had persistent fever (102.7°F, 39.3°C) and hypotension (BP 85/50 mmHg) requiring vasopressors. Surgical debridement was performed, but clinical conditions further worsened. Intra-operative samples grew a NDM-*Kp* with the same phenotypic profile as the first isolate, aside from tigecycline (MIC 1.5 mg/L) (Figure 1b). Thus, tigecycline was stopped. To evaluate susceptibility to tigecycline and eravacycline of NDM-*Kp* in our hospital, we tested 108 NDM-*Kp* isolates. Susceptibility rates to tigecycline and eravacycline were 67.6% and 64.8%, respectively. Among 37 isolates with tigecycline MIC values > 0.5 mg/L, only 2/37 (5.4%) remained susceptible to eravacycline.

In our case, eravacycline (MIC 0.25 mg/L, broth microdilution method, Thermo Scientific^™^ EUMDROXF AST Plate) 1 mg/kg q12h infused over 3 hours was added to ceftazidime/avibactam *plus* aztreonam with rapid clinical improvement.

What is the most likely explanation for this resistance profile and recommendation for treatment?

## Discussion

Metallo-β-lactamase (MBL)-producing carbapenem-resistant Enterobacterales pose a major therapeutic challenge globally.^1–4^ Unlike KPC-*Kp*, for which targeted β-lactam/β-lactamase inhibitor combinations are available, effective treatment options for MBL-*Kp* remain limited.^1,5^

To understand the resistance phenotype of NDM-*Kp*, all underlying resistance mechanisms should be analysed. Typically, NDM-*Kp* rely on multiple intrinsic and acquired determinants contributing to their multidrug-resistant profile (Table 1). Here, we discuss these mechanisms step-by-step, starting with those conferring resistance to first-line agents such as amoxicillin, and progressing to more complex mechanisms driving resistance to last-resort antibiotics.

**Table 1.** Possible resistance mechanisms in NDM-producing *K. pneumoniae* and their impact on antimicrobial susceptibility

Resistance mechanism	Type of mechanisms	Antimicrobials affected
Penicillinases, β-lactamases and carbapenemases		
*bla_SHV_*_-11_	Intrinsic	Narrow-spectrum penicillins (e.g. ampicillin)
*bla_CTX_*_-M-15_*, bla_TEM-1_, bla_TEM-32_, bla_OXA-1_, bla_OXA-9_*	Acquired	Extended-spectrum cephalosporins, third/fourth-generation cephalosporins and aztreonam
*bla_NDM_*	Acquired	Carbapenems, third/fourth-generation cephalosporins, β-lactamase inhibitor combinations
Outer membrane porins		
OmpK35 truncation	Intrinsic	Carbapenems, cephalosporins, β-lactamase inhibitor combinations; may contribute to cefiderocol resistance
OmpA/OmpU overexpression by AcrAB overexpression	Acquired	Increased susceptibility to cephalosporins and β-lactamase inhibitor combinations
Efflux pump		
AcrAB overexpression by *ramR* and *ramA* mutations	Acquired	Fluoroquinolones, tigecycline (possible reduced susceptibility to eravacycline) May restore susceptibility to β-lactamase inhibitor combinations up-regulating OmpA and OmpU
OqxAB overexpression	Intrinsic	Fluoroquinolones, reduced susceptibility to tigecycline
Other mechanisms		
*fosA*	Intrinsic	Fosfomycin (via enzymatic inactivation)
*glpT* or *uhpA* mutation	Acquired	Fosfomycin (via reduced uptake)
AAC(6′)-Ib	Acquired	Aminoglycosides (amikacin; potentially gentamicin depending on gene type)
*cirA* mutations	Acquired	Cefiderocol (high-level resistance)
*baeS* mutations, *envZ* mutations	Acquired	Cefiderocol (low-level resistance)

Although whole-genome sequencing was not performed in our NDM-*Kp*, its resistance profile can be interpreted considering the well-characterized clonal background of ST147 NDM-*Kp* circulating in the Tuscany region.^6^ It consistently carries chromosomal *bla*_SHV-11_, conferring resistance to ampicillin and amoxicillin; additional intrinsic genes such as *fosA,* and *oqxA-oqxB* can explain reduced susceptibility to fosfomycin and quinolones, respectively.^6^ Other mechanisms, including porin modifications (truncated OmpK35) have been reported in the Tuscan clone of NDM-*Kp* and contribute to the intrinsic resistance to several antibiotics, including ceftazidime.^6^

Interestingly, our strains showed increasing MIC for fosfomycin (first isolate MIC 32, second isolate MIC 128). In addition to *fosA* gene expressed into high-copy-number, acquired mutations in *glpT* or *uhpA* genes, impairing fosfomycin uptake, may have contributed to high-level fosfomycin resistance.^2,6^ Moreover, our NDM-*Kp* showed resistance to amikacin (reported in 70% of NDM-*Kp*).^1^ Acquired determinants associated with aminoglycoside resistance may include *AAC(6*′*)*.^6^  *ArmA* might also be involved in aminoglycoside resistance. However, the ArmA allelic variant Ile270Lys found in our NDM-*Kp* isolates, in the absence of other relevant aminoglycoside resistance determinants, was not associated with aminoglycoside resistance.^6^

Furthermore, acquired β-lactamases, such as *bla*_CTX-M-15_ and other determinants (*bla*_TEM-1_, *bla*_TEM-32_, *bla*_OXA-1_ and *bla*_OXA-9_) may explain resistance to several β-lactam antibiotics, including third- and fourth-generation cephalosporins and aztreonam.^2,6^ The co-production of NDM and ESBLs in our isolate is crucial to understand its resistance phenotype. In fact, aztreonam is stable against hydrolysis by MBLs, but it is usually inactivated by co-produced ESBLs. The combination ceftazidime/avibactam *plus* aztreonam (or the new antibiotic aztreonam/avibactam), recommended as first line option against MBLs, is based on this rationale: the addition of aztreonam to avibactam enhances activity by ‘protecting’ aztreonam from the ‘attack’ of ESBLs and other cephalosporinases.

Finally, up-regulation of efflux pumps, such as AcrAB (a multitransporter efflux system) and OqxAB, can also contribute to *Kp* resistance. The AcrAB-TolC pump complex extrudes compounds in an energy-dependent manner (proton antiporters) and overexpression of the AcrAB system has been associated with resistance to fluoroquinolones, β-lactams and also tigecycline. Mutations in *ramR* lead to up-regulation of RamA, which constitutively activates both AcrAB and OqxAB, raising MICs for fluoroquinolones and tigecycline.^2,6,7^ In our isolates, *ramR* and *ramA* mutations, detected in 13% of our NDM-*Kp* isolates, may have contributed to the progressive increase in tigecycline MIC.^6^ Notably, despite the increase in tigecycline MIC, our isolate remained susceptible to eravacycline. Although AcrAB overexpression has been associated with reduced eravacycline susceptibility, MICs for eravacycline are generally lower than those observed for tigecycline.^8,9^ Interestingly, AcrAB up-regulation may also enhance the expression of OmpA and OmpU, increasing outer membrane permeability and partially restoring susceptibility to β-lactamase inhibitor combinations. This synergism may support the combination of eravacycline with aztreonam/avibactam to suppress resistance emergence.^10^ Animal data demonstrated synergistic bactericidal activity of the combination of eravacycline with aztreonam/avibactam in a murine subcutaneous abscess model caused by NDM-*Kp*.^10^ Further studies are needed to confirm these observations.

Interestingly, our NDM-*Kp* isolate was resistant to cefiderocol but remained susceptible to ceftazidime/avibactam in combination with aztreonam. NDM-*Kp* isolates circulating in our region harbour a large array of acquired virulence determinants, including genes for the siderophores yersiniabactin and aerobactin, for proteins involved in iron metabolism and for several transport systems involved in the final complex resistance profile.^11^

### Conclusions

This case highlights the importance of the principles of the AMRround. The synergistic application of clinical microbiology and infectious disease expertise can support clinicians in the optimal management of difficult-to-treat cases. In this case, combination therapy enabled complete healing of the surgical wound. The role of eravacycline against NDM warrants further investigation.
